# Investigation of the Underwater Absorption and Reflection Characteristics by Using a Double-Layer Composite Metamaterial

**DOI:** 10.3390/ma16010049

**Published:** 2022-12-21

**Authors:** Yi Zhu, Xinyang Zhao, Zhiyuan Mei, Haitao Li, Dajiang Wu

**Affiliations:** College of Naval Architecture & Ocean Engineering, Naval University of Engineering, Wuhan 430000, China

**Keywords:** underwater metamaterial, acoustic absorption, acoustic reflection, NAGA-II optimization

## Abstract

It is well-known that the acoustic stealth of an underwater vehicle composed of a non-watertight structure has been facing severe challenges. The origins of this effect are associated with the fact that the coupling between the water and the mechanical structure is not negligible because both sides are in the water. Along these lines, the idea of forward absorption and backward reflection was proposed in this work to address this issue. More specifically, a composite underwater acoustic metamaterial (AM) was designed based on different layers, namely a sound absorption layer and a sound insulation layer from the outside to the inside. The sound absorption layer was made of a soft rubber matrix with embedded steel scatterers (ESs) to enrich the coupled resonance effects, while the sound insulation layer was composed of hard rubber with a built-in cavity to improve the impedance mismatching between the AM and the water. The impact of the number and thickness of the embedded ESs on the acoustic performance of the AM was also thoroughly investigated via a finite element method (FEM). A fast non-dominated genetic algorithm (NAGA-II) with elite strategy was used to optimize the position and the size of the ESs. The optimization results revealed the high absorption at the forward incidence and the high reflection at the backward incidence. Thus, our work provides a novel and effective approach for improving the acoustic stealth of underwater vehicles composed of non-watertight structures.

## 1. Introduction

The application of the anechoic coating technology is regarded as a quite important method to achieve acoustic stealth of underwater vehicles [1,2]. The reason for this effect is that not only the acoustic waves emitted by the active sonar are absorbed, but also the internal noise propagation to the outside is remarkably reduced [3,4]. In most of the reported studies [5,6,7,8,9], the subject under investigation is a watertight structure, with a relatively large impedance mismatching between the steel plate and the air. As a result, there is no specific need to consider the internal acoustic propagation to the outside in the design of the employed materials. Hence, the majority of the divulged works mainly focus on external acoustic absorption to enhance the underwater acoustic characteristics. However, the non-watertight structure of underwater vehicles on both sides of the water, as well as the water coupling effect cannot be ignored. In addition to the external acoustic absorbing, the internal acoustic propagation to the external should also be taken into account.

At present, the design of non-watertight structures intended for anechoic coatings for achieving acoustic forward absorption is still quite difficult due to the introduction of water backing, as compared to the air backing and rigid backing cases [10,11,12]. The manifestation of the water backing effect usually causes the reduction in the absorption coefficient and the absorption peak shift toward the higher frequencies. The problem of sound broadband absorption at low frequency has also not been yet fully addressed [13,14]. In many works, the incorporation of multi-layer local resonances has been proposed to broaden the absorption frequency band [15,16,17,18,19,20]. However, there are still significant difficulties due to the strong dispersion characteristics of the local resonances since the absorption bandwidth is still narrow and concentrated in the low-frequency region. Recently, the implementation of multilayer scatterers has been reported to achieve quasi-perfect absorption. Zhang et al. [21] performed an analysis from a theoretical point of view, indicating that the absorption band can be sufficiently broadened by appropriately selecting the structural parameters of the scatterers and scatterer arrays. Later, Zhang et al. [22] developed a meta-absorber based on this theoretical framework, achieving an average absorption coefficient value of 0.952 in the low frequency range of 0.5–2 kHz, which was further verified by conducting sound tube experiments. It should be noticed here that the latter work was carried out with air backing. Wang et al. [23] studied the acoustic absorption performance by using a novel two-dimensional structure with a configuration similar to that of Zhang et al. The authors utilized the FEM to analyze the local resonance characteristics and the wave scattering properties of the scatterers. On top of that, the double negativity of the structure was also investigated via the effective medium method.

Additionally, a two-layer coupling mechanism has been proposed to solve the acoustic problems of non-watertight structures. More specifically, Panigrahi et al. [24] introduced cavities with different sizes within the structure, in combination with constituting anechoic and insulation layers. The authors proposed the application of four different design solutions based on the various requirements, to reduce the echo and enhance the transmission loss performance, which is of great importance for the noise control design in underwater structures. In another interesting work, Jin et al. [4] developed a metamaterial by embedding periodic multi-resonators and voids into the anechoic and insulation layers, respectively. The authors also numerically studied the acoustic performance of the proposed two-layer structure in the water and demonstrated that the induced coupled resonance effect of the incorporated multi-resonator and voids could effectively broaden the available absorption bandwidth. However, only the forward acoustic absorption was concerned in these studies, while the impact of the backward acoustic reflection was not taken into consideration.

Several theoretical analytical methods have been also proposed to study the acoustic behavior of hydroacoustic structures. These methods include the transfer matrix method [8,25,26], the multiple scattering theory [27], the T-matrix method [28], the equivalent medium theory [29,30], etc. Nevertheless, they are mainly applied to relatively simple structures. As far as complex structures are concerned, the implementation of numerical methods, such as FEM, is more practical and flexible than the repressive analytical methods. With the constant development of computing, the scientific community began to consider structural optimization to acquire a better acoustic performance. Meng et al. [19] introduced a genetic algorithm for the optimization of the low-frequency absorption performance of a structure composed of the double-layer local resonant scatterer. Huang et al. [31] proposed a technique for the design optimization of an axisymmetric acoustic coating structure by introducing cavities to significantly improve its decoupling performance. In addition, Zhao et al. [32] used the genetic algorithm combined with the FEM to optimize the sound absorption performance of two different Alberich coatings on steel plates. From the above-mentioned acoustic optimization works, it can be argued that the employed models are based on a single objective function, which is frequently set by assigning weight to different frequency bands. Nevertheless, no multi-objective optimization has been carried out, and only the acoustic forward incidence absorption has been explored.

In view of the above, the idea of forward absorption and backward reflection was presented in this work, for the anechoic coating of the non-watertight structure to reduce the echo of external detecting sound waves, while preventing the internal sound waves propagation. A two-layer AM configuration with the incorporation of sound absorption and sound insulation layers was proposed for improving the acoustic characteristics. For the development of such types of structures, steel scatterers were embedded in the soft rubber matrix of the sound absorption layer, and the cavity was located within the structure of the hard rubber of the sound insulation layer to realize the forward absorption and the backward reflection. The accuracy of the employed finite element model was also verified by comparing the extracted outcomes with the transfer matrix method. The impact of the number of the incorporated ESs, as well as their thickness on the acoustic properties of the AM, was systematically analyzed. A two-dimensional axisymmetric simplification model was also developed, while the size of ESs and their location were optimized to improve the acoustic characteristics of the system by means of the NAGA-II algorithm [33,34].

## 2. Methodology and Modelling

### 2.1. Geometric Structure and Material

**Figure 1:**
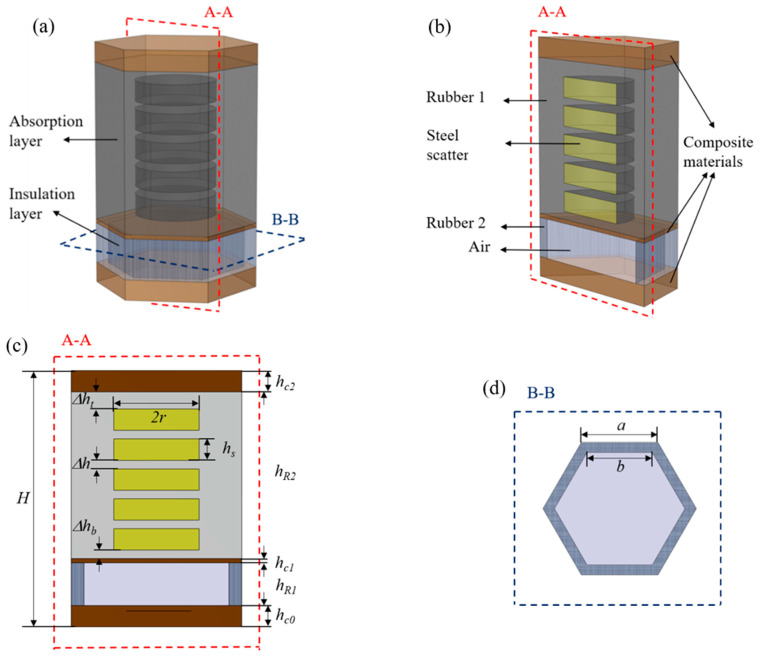
(**a**) Depiction of the hexagonal structural unit; (**b**) Schematic diagram of the structural unit; (**c**,**d**) Geometry and position distribution of each component (*H* = 60 mm, ∆*h_t_* = 4 mm, ∆*h_b_* = 2 mm, ∆*h* = 2 mm, *h_s_* = 5 mm, *r* = 10 mm, *h_c_*_0_ = 5 mm, *h_R_*_1_ = 10 mm, *h_c_*_1_ = 1 mm, *h_R_*_2_ = 39 mm, *h_c_*_2_ = 5 mm, *a* = 20 mm, *b* = 17 mm).

The hexagonal structural unit of the developed AM is shown in Figure 1a,b. It consists of a combination of a sound absorption layer, a sound insulation layer and glass fiber reinforced composites (GFRPs) with the following unit dimensions: *a* = 20 mm, *H* = 60 mm. The sound absorption layer is composed of steel cylindrical scatterers with the same thickness that are embedded in the Rubber 1 matrix to enrich the local resonance effect. In addition, the coupled resonance modes and the scattering effect between the scatterers are enhanced. The sound insulation layer comprises a honeycomb Rubber 2 and an air cavity. The outer contour shape of the honeycomb Rubber 2 is comparable with the cross-sectional area of the unit, in terms of dimensions, while its Young’s modulus is larger than that of Rubber 1, leading to the existence of a load-bearing capability. The top and bottom surfaces of the structural unit are each laminated with the incorporation of a layer of GFRPs with a thickness of *h_c_*_0_ = *h_c_*_2_ = 5 mm. An additional 1-mm-thick layer of GFRPs was also incorporated between the sound absorption and sound insulation layers to improve the overall stiffness of the proposed structure. The cross-sectional drawings of the employed structural unit, as well as the specific dimensions and locations, are depicted in Figure 1c,d. The material parameters of each component are shown in Table 1, while their influence on the frequency variation is not considered in this work.

### 2.2. Methodology and Modeling

**Figure 2:**
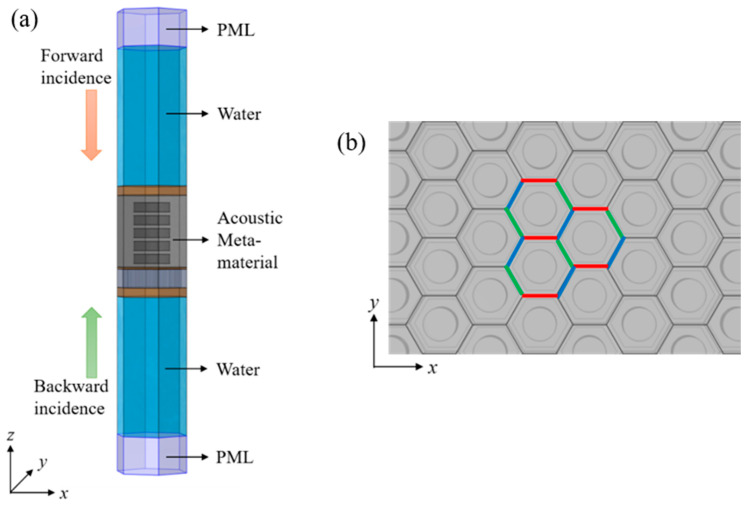
(**a**) Schematic diagram of the employed three-dimensional (3D) coordinate system, forward and backward incidence. (**b**) Depiction of the 3D spatial arrangement with periodic boundary conditions.

Both sides of the AM were regarded as infinite water (see Figure 2a). The planar acoustic waves were assumed to be incident in the *z* direction. The absorption coefficient was calculated for the forward incidence, and the reflection coefficient was considered for the backward incidence. A perfectly matched layer (PML) was also used on both sides of the water to simulate the echo-free terminal [35].

The acoustic wave in the water domain was described as follows:

(1) $$\frac{1}{c}\frac{\partial^{2}p}{\partial t^{2}}-\nabla^{2}p=0,$$

where p is the sound pressure and c represents the speed of sound in water, which is 1489 m/s.

According to the finite element method, the discrete equation can be expressed as follows:

(2) $$M_{f}\ddot{p}+K_{f}p+\rho_{0}R\ddot{u}=f,$$

where $M_{f}$ and $K_{f}$ denote the mass and stiffness matrix of the water, respectively, $\rho_{0}$ refers to the density of the water, which is 1000 kg/m^3^, p is the fluid node sound pressure, R stands for the matrix describing the coupling effect between the water and the structure domain, and f is the load applied to the water domain.

In the structure domain, the controlling equation of the employed structure can be written as follows [15]:

(3) $$M_{s}\ddot{u}+K_{s}u=F_{m}+F_{0},$$

where $M_{s}$ and $K_{s}$ are the mass matrix and the stiffness matrix of the structure, respectively, u represents the displacement vectors of the nodes of the structure, while $F_{0}$ and $F_{m}$ denote the fluid and the mechanical loads applied to the structure, respectively.

Because of the continuity of particle displacement and normal stress, the coupling surface of the fluid and the proposed structure can be described using the equation below:

(4) $$\rho_{0}\frac{\partial u}{\partial t}=-\nabla p,$$

Based on Equations (2) and (4), the discrete fluid–solid coupling equation of the structural unit can be derived as follows:

(5) $$\left[\begin{array}{cc} K_{s}-\omega^{2}M_{s} & R^{T}\\ -\rho_{0}\omega^{2}R & K_{f}-C_{\phi }-\omega^{2}M_{f} \end{array}\right]\left\{\begin{array}{c} u\\ p \end{array}\right\}=\left\{\begin{array}{c} F_{s}\\ C_{f} \end{array}\right\},$$

where $C_{\phi }$ represents the nodal value of the pressure normal gradient at the fluid boundary, and $C_{f}$ is the equivalent load applied to the fluid boundary.

The structural units are spatially arranged according to the periodicity imposed by the triangular lattice, as is shown in Figure 2b, and only one structural unit needs to be modeled and studied with respect to Bloch’s theorem [36]. The applied periodic boundary conditions can be expressed as follows:

(6) $$\Gamma \left(x+d_{x},y+d_{y},z\right)=\Gamma \left(x,y,z\right)e^{j\left(kd_{x}\sin\theta_{i}\cos\varphi_{i}x+kd_{y}\sin\theta_{i}\sin\varphi_{i}y\right.)},$$

where Γ denotes an arbitrary spatial function (either an acoustic pressure in a fluid or the displacement in a structure), $d_{x}$ and $\;d_{y}$ refer to the lattice dimensions of the single cell in the *x* and *y* directions, respectively, whereas θ (φ) represents the angle between the incident sound direction and the *z*-*(x)*axis, respectively.

By combining Equations (1), (5) and (6), the displacement u of the structure and the acoustic pressure distribution of the fluid p can be determined with a fair degree of accuracy.

Then, the sound pressure distribution at the incident and transmitted surfaces can be established as follows:

(7) $$p_{r}=\sum_{m}\sum_{n}R_{\mathrm{mn}}e^{-j\left(k_{\mathrm{mx}}x+k_{ny}y-k_{\mathrm{mn}}z\right)},$$

(8) $$p_{t}=\sum_{m}\sum_{n}T_{\mathrm{mn}}e^{-j\left(k_{\mathrm{mx}}x+k_{\mathrm{ny}}y+k_{\mathrm{mn}}z\right)},$$

where $k_{\mathrm{mx}}=\mathrm{ksin}\theta_{i}\cos\phi_{i}+\frac{m\pi }{d_{x}},k_{\mathrm{ny}}=\mathrm{ksin}\theta_{i}\sin\phi_{i}+\frac{n\pi }{d_{y}}$ and $k_{\mathrm{mn}}=\sqrt{k^{2}-k_{\mathrm{mx}}{}^{2}-k_{\mathrm{ny}}{}^{2}}$ denote the acoustic wavenumbers in *x*, *y* and *z* directions, respectively.

The reflection coefficient of each order $\;R_{\mathrm{mn}}$ and the transmission coefficient $\;T_{\mathrm{mn}}$ can be obtained by solving Equations (7) and (8). The reflection and transmission coefficients can be determined from the equations below:

(9) $$R=\sqrt{\sum_{k_{\mathrm{mn}}^{2}>0}{\left|R_{\mathrm{mn}}\right|}^{2}},$$

(10) $$T=\sqrt{\sum_{k_{\mathrm{mn}}^{2}>0}{\left|T_{\mathrm{mn}}\right|}^{2}},$$

The acoustic absorption coefficient α can be obtained as follows:

(11) $$\alpha =1-R^{2}-T^{2},$$

## 3. Results and Discussions

### 3.1. Validation of the FEM

**Figure 3:**
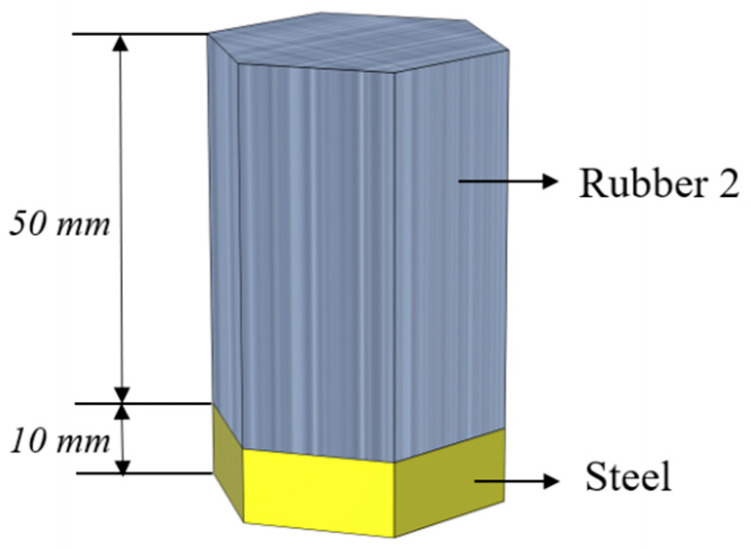
Finite element method (the verification model).

In order to verify the accuracy of the proposed numerical calculations, a 50 mm thick rubber 2 was assumed on the top of a 10 mm thick steel plate, as is shown in Figure 3. Consequently, the transmission and reflection coefficients for the cases of forward and backward incidence were calculated by employing the transfer matrix and the FEM, respectively. The finite element calculations were implemented by using the COMSOL Multiphysics finite element software. To ensure the accuracy of the calculations, a sixth of the minimum wavelength was selected for the maximum element size in the material associated with the highest concerned frequency.

**Figure 4:**
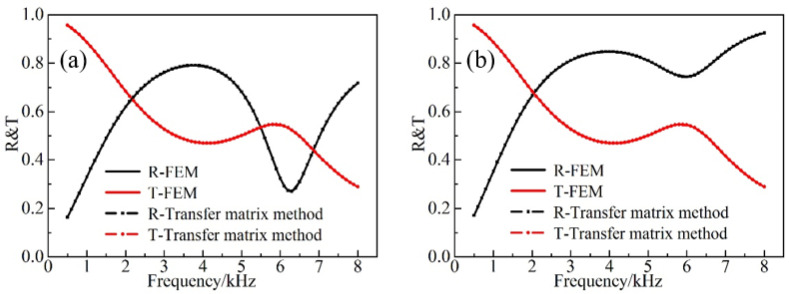
Comparison of the FEM and the analytical methods in (**a**) forward and (**b**) backward incidence.

It can be observed from Figure 4a,b, that the numerical results obtained with the FEM are in full agreement with the respective analytical data acquired via the transfer matrix method, which confirms the effectiveness of the developed FEM in this work. Owing to the reciprocity, the transmission coefficients are the same for both directions. In addition, the change of the incident order could significantly affect both the reflection and absorption characteristics of the material.

### 3.2. Acoustic Behaviors of the AM

**Figure 5:**
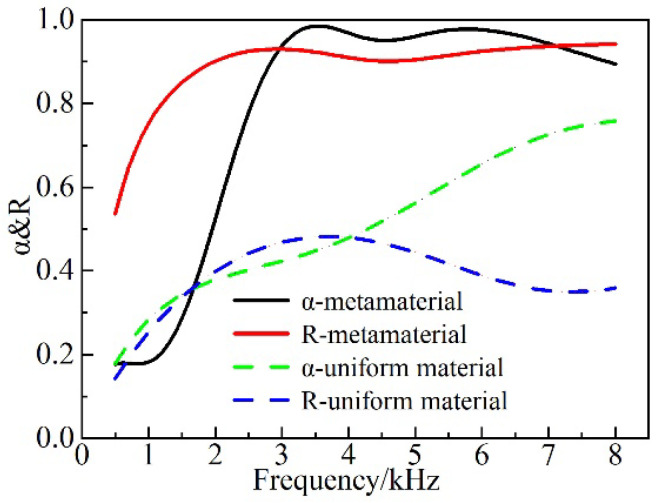
Forward absorption coefficient (α) and backward reflection coefficient (R) between the proposed AM and the uniform material.

As can be seen in Figure 5, compared with the case where a uniform material without the addition of the cavity and ESs is used, the AM can substantially and stably improve the absorption coefficient during the forward incidence relative to that of the uniform material in the frequency range of 1.7–8 kHz. Additionally, the absorption coefficient was greater than 0.8 within the frequency range of 2.6–8 kHz, whereas the sound absorption exceeded 95% in the region of 3.1–6.9 kHz, meaning that the quasi-perfect sound absorption could be achieved. At the backward incidence, the broadband reflection was realized above 1.2 kHz, and the reflection coefficient was above 0.8, i.e., being much higher than that of the uniform material.

**Figure 6:**
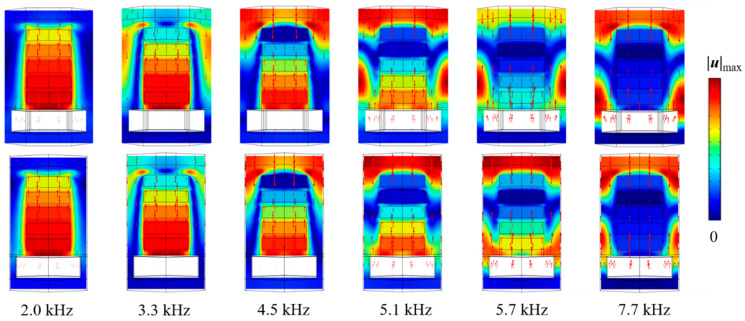
Forward absorbing displacement field and particle velocity vector (the first and second rows indicate the *x*–*z* and *y*–*z* cross-sections, respectively).

Figure 6 displays the longitudinal cross-section of the displacement field and the particle velocity vector diagram at the forward incidence. At the frequency of 2 kHz, the energy dissipation is mainly due to the resonance effect between the ESs and the cavity. The dissipation effect is limited, and the elastic wave is propagating mainly as a longitudinal wave with less loss. At 3.3 kHz, the resonance effect occurs predominately in the cavity and the bottom scatterers, while the displacement of the upper scatterers is decreased, and the angular mode appears near the middle and the upper part of the AM boundary. As far as the frequency of 5.1 kHz is concerned, the resonance effect between the cavity and the scatterers decreases. Meanwhile, the displacement of the upper scatterers continues to decrease and the angular mode moves upward, while the surface mode appears near the upper boundary. The frequency continues to increase and the surface mode disappears at the value of 5.7 kHz. At 7.7 kHz, the overall displacement of the scatterer is relatively small and the coupling between the cavity and the scatterer is nearly negligible, whereas the dissipation mainly emerges near the lateral boundary of the structural unit and in the corner region.

**Figure 7:**
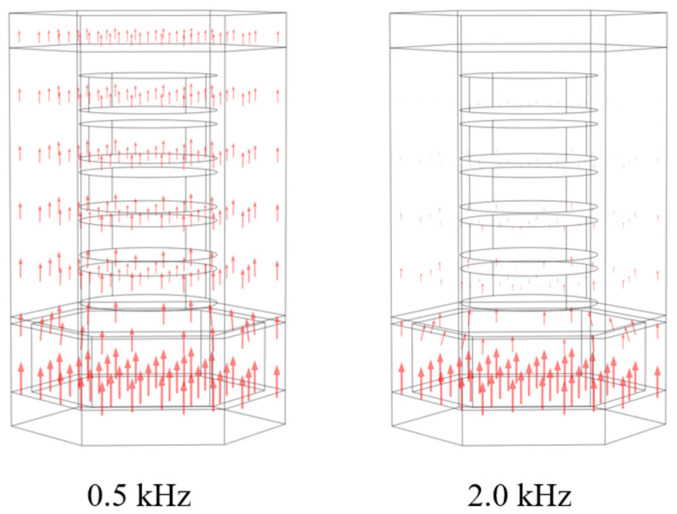
Depiction of the particle velocity vector in the backward incidence at two different frequencies.

The particle velocity vector diagram in the case of the backward incidence is shown in Figure 7. At 0.5 kHz, due to the long wavelength of the generated acoustic wave, an overall flat motion is recorded by the sound insulation and the acoustic wave enters the sound absorption layer with poor reflection characteristics. With the increase of the frequency above 2 kHz, the acoustic wave is unable to pass through the sound insulation layer owing to the impedance mismatching. As a result, the particle vibration is mainly concentrated on the surface of the cavity, and a good reflection effect can thus be achieved.

**Figure 8:**
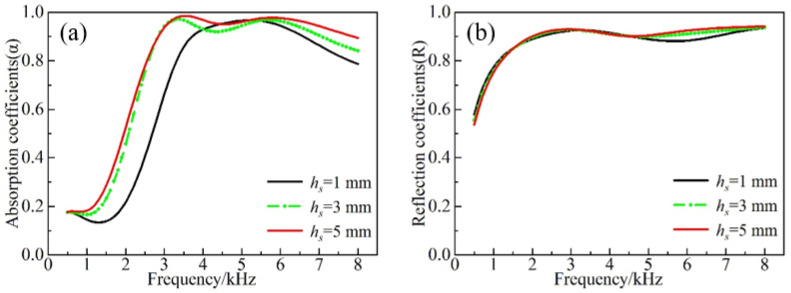
(**a**) Forward absorption and (**b**) backward reflection with different heights of scatterers.

**Figure 9:**
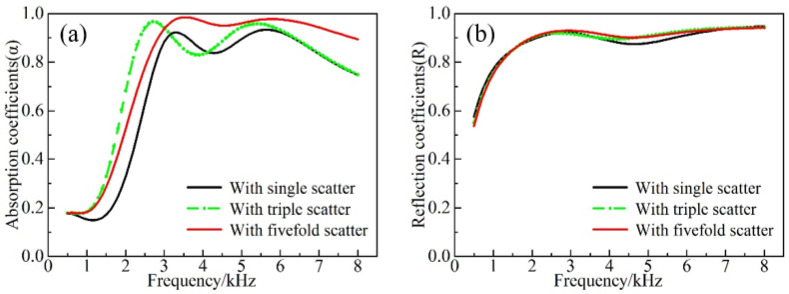
(**a**) Forward absorption and (**b**) backward reflection with different numbers of scatterers.

The proposed AMs can achieve the conceived acoustic properties mainly through the ESs, as well as the cavity, and its impact on the acoustic properties at various thicknesses and quantities of ESs is shown in Figure 8 and Figure 9.

According to Figure 8a, the first absorption peak at the forward incidence shifted forward and increased as the thickness of the ESs became larger. Moreover, at the frequency above 5.5 kHz, the increase in the thickness improved the acoustic absorption characteristics. Meanwhile, the difference between *h_s_* = 3 mm and *h_s_* = 5 mm was not significant at the frequency below 3.1 kHz. This is due to the fact that various ESs layers interacted with each other at higher frequencies to absorb the sound. Hence, the difference between the three cases became more obvious with increasing frequency. At low frequencies, the synergistic effect of the bottom scatterers and the cavity induced the formation of a characteristic mode to effectively dissipate the sound energy. However, the corresponding angular mode cannot be formed for the scatterer with a thickness of *h_s_* = 1 mm, which is due to its relatively small mass. Hence, the low frequency acoustic absorption is relatively poor. As can be observed from Figure 9a, the absorption effect in the forward incidence gradually increased with the increase in the number of scatterers at the higher frequency (>4.2 kHz) at forward incidence. The implementation of a fivefold scatter yielded better absorption characteristics than the single scatter in the whole frequency band. The first absorption peak of the triple scatter was shifted to the frequency of 2.7 kHz, and the absorption coefficient reached the value of 0.97. The absorption coefficient was slightly reduced at the frequency range of 3–4.5 kHz. The origins of this effect were associated with the fact that the upper scatterers in the fivefold scatter can effectively suppress the dissipative effect between the bottom scatterers and the cavity. As seen from Figure 8b and Figure 9b, the thickness and the number of scatterers at the backward incidence have a negligible impact on the reflection.

## 4. Model Simplification and Optimal Design

### 4.1. Model Simplification

**Figure 10:**
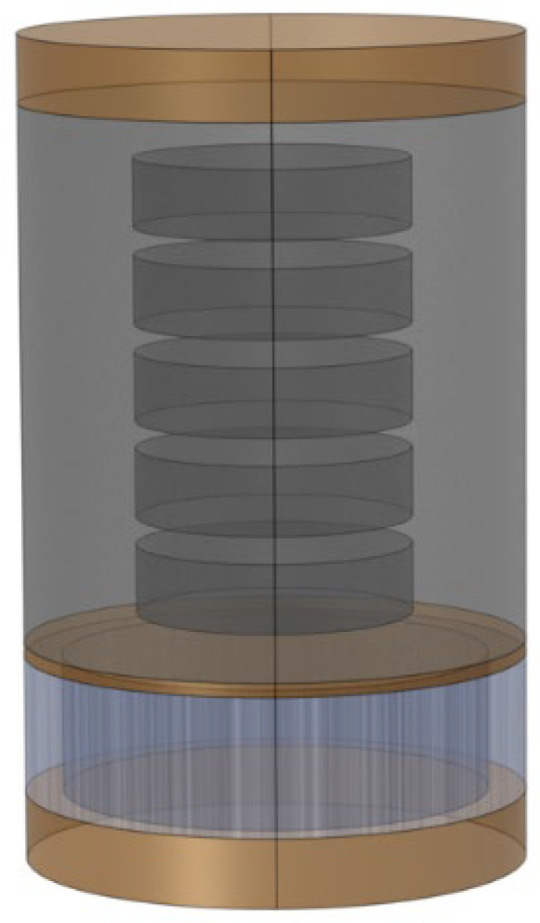
Simplified axisym 2D model.

Due to the relatively low transverse wave speed of the rubber, a large computational volume was induced by meshing, which is not conducive to the optimization of the structure. By considering the transformation of the 3D configuration into a two-dimensional axisymmetric (axisym 2D) configuration [37] in the case of normal incidence, the periodic boundary condition can be simplified to u•n=0. In turn, the original hexagonal structural unit can be also simplified to an axisym 2D unit in assuming the constant volume share of each component, as can be ascertained from Figure 10.

**Figure 11:**
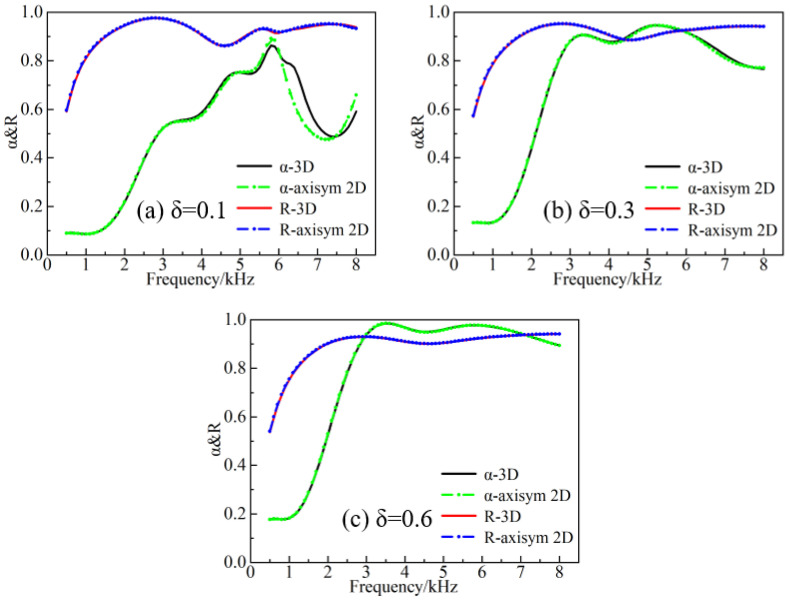
Forward absorption and backward reflection with different loss factors.

It was pointed out in Ref. [38] that the loss factor is regarded as an important factor affecting the simplification accuracy. However, it is worth mentioning that only the cavity-containing configuration has been studied in this work and needs to be reconsidered for specific configurations. In Ref. [22], the impact of the loss factor on the acoustic absorption was investigated, but the backward reflection property was not addressed. In addition, since the GFRPs exhibit an orthogonal anisotropy, the accuracy of calculations should be contemplated regardless of the fact that the reduction of the axisym 2D can significantly affect the simulated outcomes. Figure 11 displays the impact of changing the loss factor of rubber 1 between the 3D and the simplified configurations, at the forward and backward incidence. As can be observed from Figure 11, the 3D and the simplified configurations are in direct agreement with each other in the forward absorption curve at the frequencies between 0.5 and 5.5 kHz and the loss factor δ = 0.1, while the difference gradually becomes apparent at the higher frequency. This effect may be due to the role of the modes formed near the lateral boundary and the corner region with increasing frequency, while the influence of the corner part in the simplified structure is omitted. By further increasing the loss factor, the overall sound absorption curve is stabilized, and the absorption coefficient is increased. The main mechanism of the backward reflection lies in the impedance mismatch of the air cavity and the water. For that reason, the volume share of the air cavity is kept constant during the simplification process. Thus, the change of the loss factor and the simplification of the configuration do not affect dramatically the previous process. At the loss factor value of δ = 0.6, the calculated curves matched perfectly, and the influence of the material parameters of the GFRPs could be disregarded at this time.

### 4.2. Optimization

Based on the validated axisym 2D model, the NAGA-II algorithm also served for optimizing the design of the ESs in the AM. The NAGA-II algorithm is considered a fast non-dominated genetic algorithm with elite strategy. Underlying the Pareto optimal solution scheme, it is also commonly applied to solve multi-objective optimization problems. In this study, three improvements were made using the genetic algorithm to balance the relationship between various objective functions. First, the complexity of the non-dominated ranking method could be reduced from mN^3^ to mN^2^ (here, m is the number of the optimization objectives, and N represents the number of populations). Second, the elite strategy was introduced, which ensured that the good individuals would not be discarded during the evolution. Third, the crowding degree and crowding degree comparison operator were introduced, thus providing the diversity of the population. For the optimization problem of the forward absorption and backward reflection, the specific procedure employed in present work is as follows.

(1) The thickness, radii and spacings of the five different scatterers embedded in the ruber1 matrix were numbered sequentially from the bottom to the top, e.g., the first scatterer thickness was *h_s_*_1_, the radius was *r*_1_, and the spacing between the first and second scatterer was ∆*h_s_*_12_. The specific control parameter ranges are shown in Table 2. Meanwhile, no attempt was made to optimize the existing material parameters. The optimization process was carried out by imposing a constraint on the equation, to ensure that the thickness of the sound absorption layer was constant.
  (12) $$\sum_{l=1}^{5}h_{sl}+\sum_{i=1}^{4}∆h_{ij}+∆h_{t}+∆h_{b}=h_{R2},\;\;j=i+1,$$
(2) The optimized frequency band range was [0.1–8 kHz] with a frequency step ∆f = 0.1 kHz. The main parameters of the genetic algorithm were set as follows: the number of evolutions was 50, the population size was 90, the probability of variation was 0.8, and the probability of crossover was 0.8.
  The objective function was defined by considering the forward absorption and backward reflection, while the overall optimization in the frequency band was taken into account to avoid the situation where the average absorption coefficient could reach the objective function. However, the acquired curve greatly fluctuated. As it could be seen from Figure 5, the absorption coefficient was low in the frequency range of 0.1–2 kHz. Therefore, the absorption band was divided into two bands to prevent the interference of this band with the averaging frequency range of 2.1–8 kHz. Moreover, the scatterer size and position had less influence on the acoustic reflection, and the frequency band was not divided during the optimization process.
  The corresponding optimization objective function is as follows:
  (13) $$\left\{\begin{array}{c} Obj1=min\left(-ave\left(\alpha \right)\right),0.1\;\mathrm{kHz}\leq f\leq 2\;\mathrm{kHz}\\ Obj2=min\left(-ave\left(\alpha \right)\right),2.1\;\mathrm{kHz}\leq f\leq 8\;\mathrm{kHz},\\ Obj3=min\left(-ave\left(R\right)\right),0.1\;\mathrm{kHz}\leq f\leq 8\;\mathrm{kHz} \end{array}\right.$$
(3) Population initialization consisted in randomly generating the primitive population according to the population size, while each individual in the population consisted of 16 model parameters.
(4) The ranking populations were generated according to the dominance and non-dominance relationships between the individuals in the population and by classifying the populations by their class.
(5) The dominant individuals were selected according to the optimization objectives and subpopulations were generated produced after performing crossover and mutation operations.
(6) During the beginning of the second generation, the parent population was merged with the child population. Then, the non-dominated sorting operation was executed successively, while the crowding degree was calculated for the individuals in each level.
(7) The dominant individuals were selected based on population stratification and individual crowding, and crossover and mutation operations were performed.
(8) The final step was to determine whether the termination condition was reached. If yes, the output of the Pareto optimal solution was set, otherwise the steps (6)–(8) were repeated.

**Figure 12:**
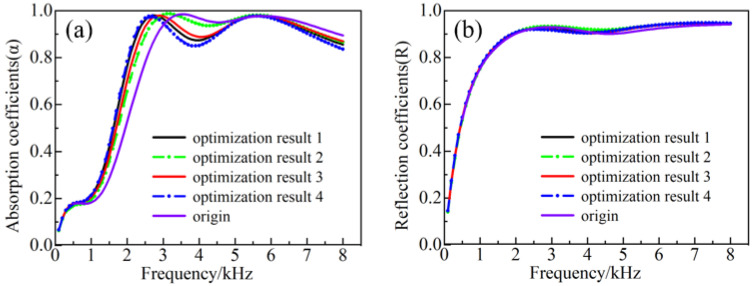
Optimization results for (**a**) forward absorption and (**b**) backward reflection.

The optimized parameters are shown in Table 2. Figure 12 depicts the acquired results of the forward absorption and backward reflection coefficients for some of the optimized model parameters. As can be seen from Figure 12, there were distinct differences in the optimization results, mainly at the frequencies of 2 kHz and 4 kHz, where a valley was detected. From the optimization result 1, a forward absorption coefficient of 0.76 was achieved at 2 kHz, which could significantly improve the absorption performance between the frequencies of 2 and 3 kHz. On top of that, the absorption coefficients at the frequencies of 2.1 to 8 kHz are greater than 0.8, with an average absorption coefficient of 0.93. The backward reflection coefficients are higher than the values of 0.8 and 0.9 within the frequency ranges of 1.2 to 8 kHz and 1.9 to 8 kHz, respectively.

## 5. Conclusions

Underwater AM has experienced tremendous growth in recent years due to the increasing interest in the acoustic stealth performance of underwater vehicles. However, for the anechoic coating of the non-watertight structure, the design of underwater AM still faces significant challenges because of the existence of a non-negligible coupling effect between the structure and water. Given that the sound waves will be incident in two directions, conventional acoustic absorption strategies have been realized to make the AM mass larger owing to the need to consider the impedance matching between the AM and the water, which is not conducive to the use of underwater vehicles. A stealth strategy of forward acoustic absorption and backward acoustic reflection was presented in the present work. Under this perspective, a two-layer structured underwater AM with sound absorption and sound insulation layers was proposed, in which steel scatterers were embedded in the soft rubber matrix of the sound absorption layer and the cavity was arranged in the hard rubber of the sound insulation layer. The scattering and the coupling resonance effects between the scatterers in the sound absorbing layer, as well as between the scatterers and the cavity, led to a variety of eigenmodes with high energy loss and the generation of high acoustic absorption. Changing the number and thickness of ESs affected the formation of eigenmodes, which had an effect on the acoustic absorption performance. More specifically, the increase in the thickness of the ESs could broaden the acoustic absorption in the low-frequency range, while exerting a negligible impact on the backward reflection caused by the impedance mismatching between the AM and the water. Afterward, the NAGA-II algorithm could solve the multi-objective optimization problem with forward high absorption and backward high reflection by optimizing the size and position of the scatterers. The absorption coefficient was 0.76 at the frequency of 2 kHz in the forward incidence but increased above 0.8 at the frequencies of 2.1 to 8 kHz with an average value of 0.93. The reflection coefficient was greater than 0.8 at the frequencies of 1.2 to 8 kHz and over 0.9 in the range of 1.9 to 8 kHz at the backward incidence, respectively.

Finally, it should be noted that the main concept of present study focus was the application of novel ideas and solutions for the both-side consideration of the acoustical design of non-watertight structures, without conducting the corresponding experimental studies. Since the underwater environment is complex, the anechoic coating may be deformed and the material properties may be significantly affected by the hydrostatic pressure, while the acoustic properties should be further examined in future works.

## Figures and Tables

**Table 1:** The property parameters of the materials.

	Density (kg/m^3^)	Young’s Modulus (Pa)	Shear Modulus (Pa)	Poisson’s Ratio	Loss Factor
Composite materials	1950	{1.6 × 10^10^, 1.6 × 10^10^, 2.9 × 10^9^}	{7.5 × 10^9^, 4.5 × 10^9^, 4.5 × 10^9^}	{0.14, 0.25, 0.25}	0.01
Rubber1	1100	2.7 × 10^7^	-	0.49	0.6
Steel	7870	2 × 10^11^	-	0.29	0.01
Rubber2	1100	1 × 10^8^	-	0.45	0.1

**Table 2:** Model optimization parameters.

	*h_s_*_1_	*h_s_*_2_	*h_s_*_3_	*h_s_*_4_	*h_s_*_5_	*r*_1_	*r*_2_	*r*_3_	*r*_4_	*r*_5_
Lower limit	0.5	0.5	0.5	0.5	0.5	1	1	1	1	1
Upper limit	8	8	8	8	8	14	14	14	14	14
Optimization result 1	4	8	8	8	8	6	10	10.2	9.9	1
	∆*h_b_*			∆*h_s_*_12_	∆*h_s_*_23_	∆*h_s_*_34_	∆*h_s_*_45_	∆*h_t_*		
Lower limit	0.5			0.5	0.5	0.5	0.5	0.5		
Upper limit	4			4	4	4	4	4		
Optimization result 1	0.5			0.5	0.5	0.5	0.5	0.5		

## Data Availability

Not applicable.
